# Decoding the secrets: how conformational and structural regulators inhibit the human 20S proteasome

**DOI:** 10.3389/fchem.2023.1322628

**Published:** 2024-01-08

**Authors:** Pedro M. P. Fernandes, Romina A. Guedes, Bruno L. Victor, Jorge A. R. Salvador, Rita C. Guedes

**Affiliations:** ^1^ Laboratory of Pharmaceutical Chemistry, Faculty of Pharmacy, University of Coimbra, Coimbra, Portugal; ^2^ Center for Innovative Biomedicine and Biotechnology (CIBB), Center for Neuroscience and Cell Biology (CNC), University of Coimbra, Coimbra, Portugal; ^3^ Research Institute for Medicines (iMed.ULisboa), Faculdade de Farmácia, Universidade de Lisboa, Lisboa, Portugal; ^4^ BioISI─Biosystems & Integrative Sciences Institute, Faculty of Sciences, Universidade de Lisboa, Lisboa, Portugal

**Keywords:** 20S proteasome inhibitors, drug resistance, mutations, molecular dynamics, molecular docking

## Abstract

Acquired resistance to drugs that modulate specific protein functions, such as the human proteasome, presents a significant challenge in targeted therapies. This underscores the importance of devising new methodologies to predict drug binding and potential resistance due to specific protein mutations. In this work, we conducted an extensive computational analysis to ascertain the effects of selected mutations (Ala49Thr, Ala50Val, and Cys52Phe) within the active site of the human proteasome. Specifically, we sought to understand how these mutations might disrupt protein function either by altering protein stability or by impeding interactions with a clinical administered drug. Leveraging molecular dynamics simulations and molecular docking calculations, we assessed the effect of these mutations on protein stability and ligand affinity. Notably, our results indicate that the Cys52Phe mutation critically impacts protein-ligand binding, providing valuable insights into potential proteasome inhibitor resistance.

## 1 Introduction

**FIGURE 1:**
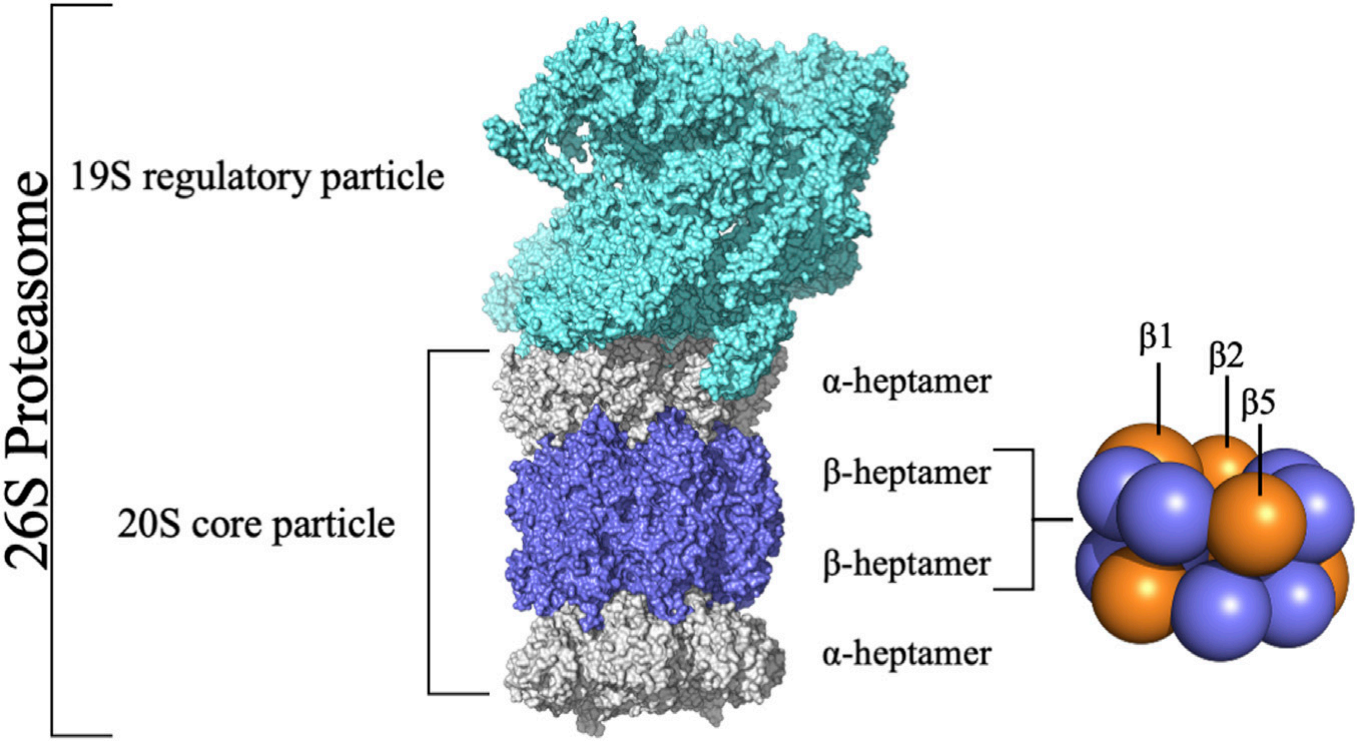
Structure of the 26S Proteasome. The 26S proteasome comprises the 20S core particle capped by the 19S regulatory particle. The 20S core particle comprises 28 subunits grouped in four rings stacked into a α-β-β-α pattern.

Cellular homeostasis is a tightly controlled process balancing protein synthesis and degradation mechanisms (Chondrogianni et al., 2015). In eukaryotic cells, intracellular protein degradation primarily occurs through two pathways: lysosomes and the Ubiquitin-Proteasome Pathway (UPP), also known as the Ubiquitin-Proteasome System (UPS). The UPS is crucial in ATP-dependent protein degradation within the cytoplasm and nucleus, affecting cell cycle control, apoptosis, DNA repair, transcription, immune response, and signaling processes by degrading key cellular players like cyclins and tumor suppressors (Hochstrasser, 1995; Ciechanover, 2007). Dysfunctions in these pathways are linked to diseases like cancer and neurodegeneration (Kisselev et al., 2003; Da Fonseca and Morris, 2008; Da Fonseca et al., 2012; Schweitzer et al., 2016). Central to the UPS is the 20S core particle (or 20S proteasome) (Figure 1), responsible for degrading unnecessary or damaged proteins, facilitated by its catalytic subunits (Ciechanover, 2007; Finley, 2009; Blackburn et al., 2010; Verbrugge et al., 2015). Structurally, the 20S proteasome consists of a cylindrical assembly of approximately 160 Å in length and 120 Å in diameter, formed by four heptameric rings (two α-rings and two β-rings in an α-β-β-α arrangement) (de Bettignies and Coux, 2010; Jung and Grune, 2012).

**FIGURE 2:**
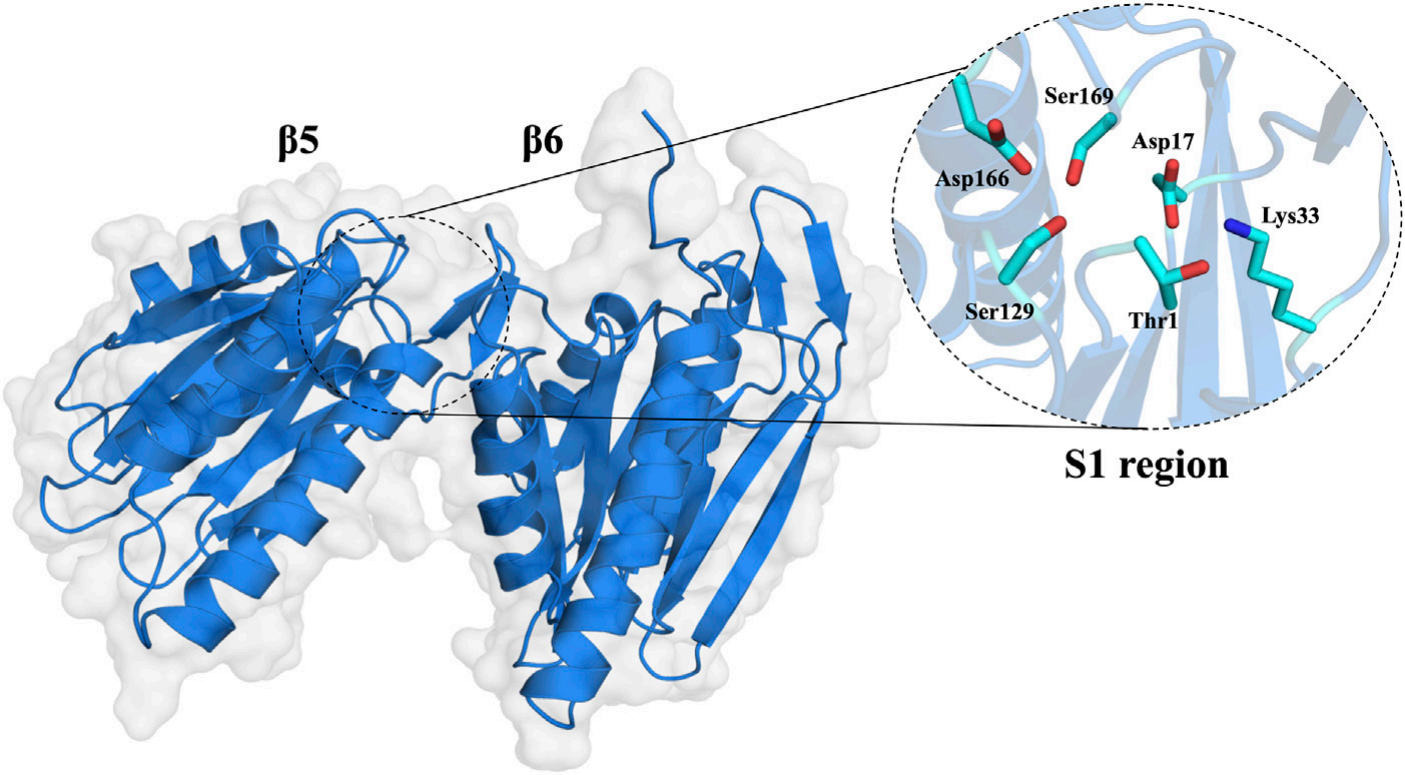
Molecular surface of the 20S proteasome β5 and β6 subunits and zoom of the CT-L catalytic site of the 20S proteasome (S1 region).

**FIGURE 3:**
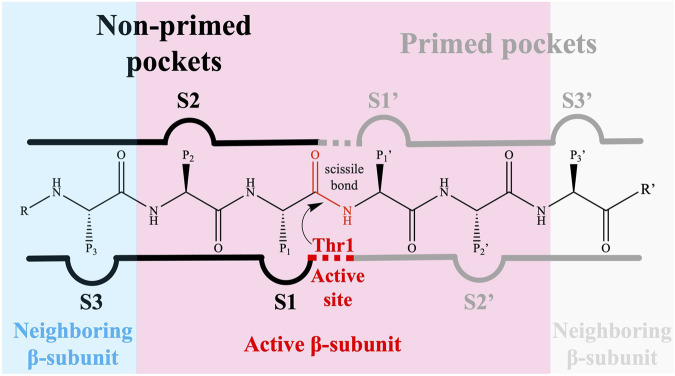
The proteasomal substrate binding channel with non-primed (S) and primed (S′) specificity pockets interacting with the amino acid side chains (P-sites) of a peptide. The reactive subunit, which contains the catalytic Thr1, is responsible for the primed substrate binding channel as well as the non-primed S1, S2, and S3 pockets.

The 20S proteasome’s multiple catalytic sites, β1, β2, and β5, each with unique specificities, enable efficient degradation of cellular proteins. While inhibiting β1 or β2 does not significantly impact protein degradation, targeting β5 dramatically reduces it. Each of these sites features an N-terminal threonine (Kisselev et al., 2006) which, through its γ-hydroxyl moiety, acts as a nucleophile in peptide bonds hydrolysis (Zhu et al., 2009; Diez-Rivero et al., 2010; Beck et al., 2012). The substrate-binding sites share a topology where the S1 region is buried in the subunit adjacent to the threonine, S2 is exposed, and both the catalytic unit and its neighbor contribute to the S3 position (Groll and Huber, 2003). These subunits demonstrate distinct cleavage preferences: β1 shows “caspase-like” (C-L) or “post acidic” (PA) activity, β2 has “trypsin-like” (T-L) activity, and β5 exhibits “chymotrypsin-like” (CT-L) activity (Nussbaum et al., 1998; Groll et al., 1999; Kisselev et al., 1999; Borissenko and Groll, 2007; Basse et al., 2010; Beck et al., 2012; Huber et al., 2012). Although catalytic activity occurs only at β1, β2, and β5 subunits, the contribution of adjacent subunits significantly impacts the definition of the catalytic pockets, substrate stabilization, and positioning (Loizidou and Zeinalipour-Yazdi, 2014). Key amino acids at these sites, especially threonine 1 (Thr1), aspartate 17 (Asp17), lysine 33 (Lys33), serine 129 (Ser129), aspartate 166 (Asp166), and serine 169 (Ser169) (Figure 2), play pivotal roles in both catalysis and maintaining the structural activity of the active site (Unno et al., 2002; Borissenko and Groll, 2007; Huber et al., 2012). Despite the protonation of Thr1 N-Terminal (Thr1N) under physiological conditions, which makes it unlikely initial nucleophile (Trivella et al., 2014), its Oγ atom is considered the general nucleophile in proteasome interactions. The other residues (Ser129, Asp166, and Ser169) contribute not only to catalysis but also to the structural integrity of the active site (Figure 2) (Unno et al., 2002; Borissenko and Groll, 2007; Huber et al., 2012). The substrate binding channels of the proteasome exhibit distinct primed and non-primed specificity pockets, facilitating the binding of target polypeptides in a C- to N-terminal direction (Figure 3). The primed sites, characterized by shallow profiles, likely facilitate the early release of the C-terminal cleavage product within the reaction cycle. In contrast, the pronounced non-primed pockets enable tight interactions with the N-terminal polypeptide segment, thereby substantially influencing cleavage specificity (Groll et al., 1997). The scissile peptide bond, positioned between primed and non-primed sites, undergoes cleavage catalyzed by the active site Thr1 residue (Figure 3), thereby classifying the proteasome as an N-terminal nucleophile hydrolase. Harshbarger et al. (Harshbarger et al., 2015) described that the substrate selectivity for each active site is determined by the interaction of the P1 side chain of the substrate with the S1 specificity pocket of the active site. Residue 45 at the base of the S1 pocket is essential to determine the three different cleavage preferences; in the CT-L active site, the β5 subunit amino acids of the S1 pocket responsible for the CT-L activity are Ala20, Met45, Ala49, and Cys52. Met45 protrudes from the pocket, allowing it to accommodate hydrophobic residues such as alanine, valine, or tyrosine (Figure 3) (Harshbarger et al., 2015). The mutation of these residues, especially in the S1 pocket, impacts drug binding and thus resistance mechanisms, highlighting the need for understanding their structural and functional roles for developing efficient proteasome inhibitors.

### 1.1 Proteasome inhibitors and drug resistance

**FIGURE 4:**
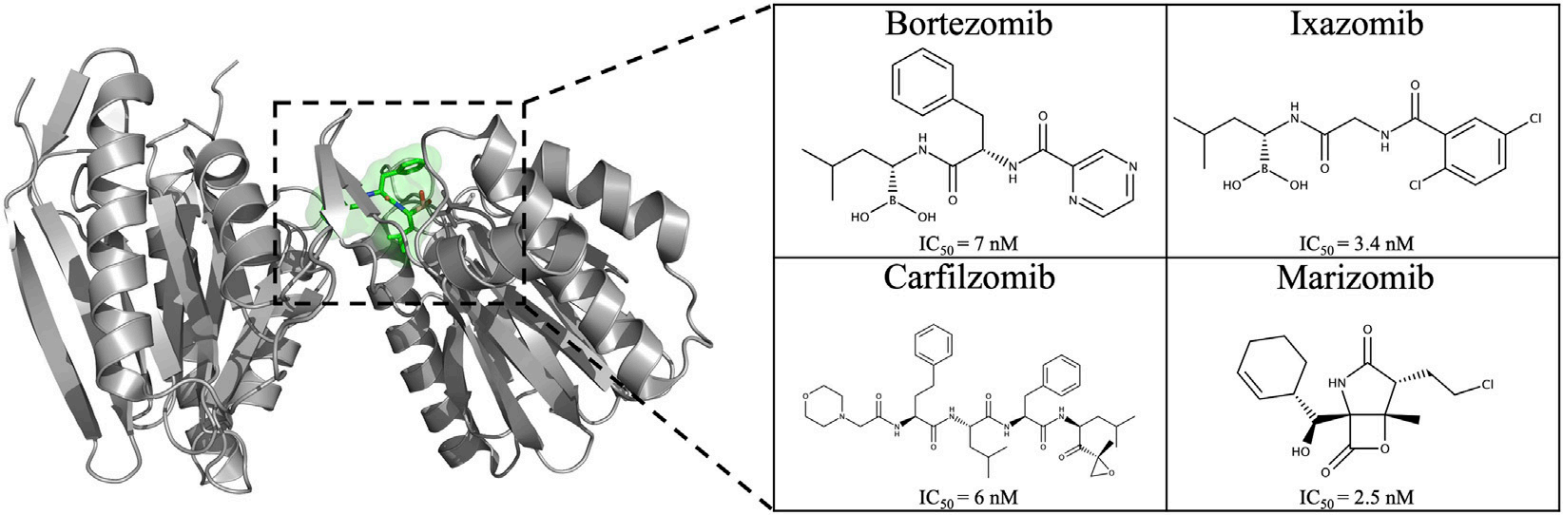
Chemical structure of the four proteasome inhibitors currently on the market and/or approved. IC_50_ values of PIs on the CT-L active site: bortezomib (Demo et al., 2007), carfilzomib (Demo et al., 2007), ixazomib (Kupperman et al., 2010), and marizomib (Chauhan et al., 2005).

The development of 20S proteasome inhibitors (PIs) has been a pivotal strategy in treating diseases associated with the UPS. Various PIs, including peptide aldehydes, boronates, α′,β′-epoxyketones, and others, have been identified (Kisselev et al., 2012; Micale et al., 2014). Bortezomib (2003), carfilzomib (2012), and ixazomib (2015) are FDA-approved PIs (Figure 4) for treating refractory multiple myeloma (MM) and mantle cell lymphoma (Kortuem and Stewart, 2013; Kisselev and Groettrup, 2014; Merin and Kelly, 2015; Teicher and Tomaszewski, 2015; Shirley, 2016), with marizomib (Figure 4) still under clinical trials. In 2013, FDA approved marizomib as an orphan drug for the treatment of MM and in 2015 for malignant glioma (European Medicines Agency, 2023; FDA, 2023).

Resistance to PIs, both innate and acquired, is a significant challenge in therapy, as underscored by Leonardo-Sousa et al. (2022). Efforts to tackle this issue have largely focused on the covalent reversible inhibitor bortezomib (Kale and Moore, 2012; Petrucci et al., 2013; Schmitt et al., 2014). While bortezomib initially elicits favorable responses in many patients, resistance often develops, leading to treatment failure or relapse (Lü et al., 2008a; Lü et al., 2008b; Franke et al., 2012; Manasanch et al., 2014).

The development of resistance to PIs is often linked to mutations in target proteins, a well-established phenomenon in cancer drug resistance. Studies by Kale and Moore (2012) and Niewerth et al. (2015) highlight the strong association between acquired resistance to PIs and point mutations in PSMB5. These mutations, occurring either near or distal to the PSMB5 binding site, can alter drug affinity by either preventing compound binding or inducing allosteric inhibition. Several other groups (Lü et al., 2008a; Lü et al., 2008b; Lü et al., 2009; Franke et al., 2012; Lichter et al., 2012; Manasanch and Orlowski, 2017) have found that continued *in vitro* exposure of cell lines related to hematological cancers (e.g., RPMI8226, CCRF-CEM, and Jurkat cells) to the proteasome inhibitor bortezomib may lead to mutations in the PMSB5 gene (which encodes the β5 subunit of the 20S proteasome), causing different point mutations, e.g., Ala49Thr, Ala50Val, Thr21Ala, Met45Val, and Cys52Phe. Franke et al. (2012) even mention the existence of a “mutation cluster region”, describing that most mutations occur around the highly conserved S1 pocket of the β5 subunit (Figure 3) (Lü et al., 2008a Lü et al., 2008b; Lü et al., 2009; Oerlemans et al., 2008; Ri et al., 2010; Franke et al., 2012; Verbrugge et al., 2012). These mutations, including Ala49Thr and Cys52Phe, often cluster around the S1 pocket of the β5 subunit, affecting bortezomib binding and catalytic activity by altering hydrogen bond formation and causing steric hindrance. This mutation-induced alteration in the proteasome’s structure, particularly in the S1 pocket, underscores the complexity of overcoming resistance in therapeutic approaches targeting the proteasome (Figure 3) (Groll et al., 2006; Franke et al., 2012; Huber et al., 2012).

Considering the primary targeting of the β5 subunit by PIs, and the occurrence of mutations in this subunit, understanding their role in resistance mechanisms is crucial for developing effective inhibitors (Kale and Moore, 2012; Niewerth et al., 2015).

This study focuses on the significant mutations in the β5 subunit, investigating how these alterations impact proteasome structure and interaction with inhibitors. Utilizing molecular dynamics and docking methods (Guedes et al., 2016; Guedes et al., 2023), we conducted a detailed analysis of various point mutations near the binding site. Our goal is to comprehend how these mutations influence resistance to bortezomib, comparing mutated proteasome structures with their wild type (WT) counterparts. The insights from this research will inform strategies for designing drugs that can effectively target these mutations, enhancing the fight against drug resistance (Guedes et al., 2019; Guedes et al., 2023).

## 2 Methods section

### 2.1 System setup

In this work, we performed molecular dynamics (MD) simulations for the two β-rings (each composed of β1-7 subunits) of the 20S proteasome core particle. Four systems were simulated: native WT, Ala49Thr, Ala50Val, and Cys52Phe mutants. The initial coordinates for 20S proteasome simulations were obtained from the X-ray structure with PDB code 5LE5 (Schrader et al., 2016) and were prepared by stripping out any water molecules, ions, and ligands.

### 2.2 Modeling of mutants

Three mutants (Ala49Thr, Ala50Val, and Cys52Phe) were prepared from the native WT (PDB code: 5LE5), using the mutagenesis wizard module of PyMOL software (Schrödinger, 2023) and applied to both β5 subunits (one in each β-ring).

### 2.3 Molecular dynamics simulations

All simulations were performed using the GROMACS 2016.3 (Berendsen et al., 1995; Lindahl et al., 2001; Van Der Spoel et al., 2005; Hess et al., 2008; Pronk et al., 2013; Abraham et al., 2015; Páll et al., 2015) software package and the GROMOS 54a7 (Schmid et al., 2011) force field together with the SPC water model (Berendsen et al., 1981). The protonation states of the titratable protein residues were set using the GROMACS pdb2gmx tool, taking into account their dominant form at pH 7.4. The N- and C-terminals from all subunits were set to their charged form. The Thr1 residue at the binding site was protonated in agreement with the observations in the X-ray diffraction study performed by Schrader et al. (2016). In each simulated system, the protein (in its WT and mutant forms) was solvated in a dodecahedron simulation box and then neutralized by adding 14 Na+ ions. Each system was first minimized with 1,000 steps of steepest descent, followed by 1,000 steps of conjugated gradient. After minimization, the system was equilibrated by performing 1 ns in the canonical (NVT) ensemble using the V-rescale weak-coupling method (Bussi et al., 2007) followed by 1 ns in the NPT ensemble with the Parrinello-Rahman barostat (pressure 1 bar) (Parrinello, 1981; Nosé and Klein, 1983). During this process, the heavy atoms of the protein were restrained using a constant force of 1,000 kJ/mol/nm^2^ After the equilibration process, we performed three replicate simulations for each simulated system, each one 100 ns long. In these production runs, the conformational space was sampled according to the NPT ensemble with the pressure set to 1 bar with a coupling constant of 2 ps (Parrinello-Rahman barostat) and a temperature set at 300 K using a coupling constant of 0.1 ps (V-rescale weak coupling). An isotropic pressure coupling with compressibility of 4.5 × 10^−5^ bar^-1^ was used. The long-range electrostatics were calculated with the particle mesh Ewald (Darden et al., 1993; Essmann et al., 1995) method, with a real space cutoff of 1.0 nm and a Fourier grid spacing of 0.12 nm. Van der Waals interactions were truncated above 1.0 nm. All protein bonds were constrained using the LINCS algorithm while SETTLE was used to constrain the water molecules. The equations of motion were integrated every 2 fs with an update of the neighbor’s list done every 10 steps.

### 2.4 MD simulation analyses

All MD simulations were analyzed using rms, gyrate, hbond, and distance tools implemented in the GROMACS software package (Van Der Spoel et al., 2005). These utilities allowed us to obtain the root-mean-square deviation (RMSD), the radius of gyration (Rg), the number of hydrogen bonds (H-bonds), and the distance between residues, respectively. The open-source POVME (Durrant et al., 2011; 2014; Wagner et al., 2017) binding pocket analysis software was used to calculate the catalytic pocket volume of the β5 subunit, which maps the flexibility of the binding pocket employing a voxel/grid-based 3D pocket representation.

All the molecular graphical presentations were created using PyMOL (Schrödinger, 2023) and all plots were generated using Gnuplot (Williams et al., 2019).

### 2.5 Molecular docking

Covalent docking calculations of the proteasome inhibitor bortezomib were performed using GOLD 2020.1 (Jones et al., 1997). Bortezomib protonation states were initially determined at pH = 7.4 and 300 K. Partial atomic charges were assigned using the Amber10:EHT force field implemented in the Molecular Operating Environment (MOE) 2019.0102 software package (Chemical Computing group ULC, 2023). After parameterization, all compounds were energy-minimized. The genetic algorithm implemented in GOLD was used to generate different ligand interaction binding poses. The generated solutions were then ranked using the CHEMPLP (Korb et al., 2009) scoring function.

The docking calculations were performed using the human proteasome crystal structure available on PDB (PDB code: 5LF3) and on four different MD-derived structures of the WT, the Ala49Thr, Ala50Val, and Cys52Phe mutants (β5 subunit). The structures represent the central conformation of the equilibrated region of all MD replicate simulations. The human proteasome 3D structure (PDB code: 5LF3 – 20S proteasome complexed with bortezomib (Schrader et al., 2016)) was prepared using MOE 2019.0102 (Chemical Computing group ULC, 2023). All calculations were performed on the CT-L binding site of the β5-subunit. Hydrogen atoms were added, and the protonation states of the side chains of the protein residues were the same as previously used in MD simulations. The AMBER10:EHT force field was then used to assign atom types and charges to each atom in the receptor. The Thr1 residue at the binding site was protonated in agreement with the observations in the X-ray diffraction study performed by Schrader et al. (Schrader et al., 2016). The “searching space” in all docking calculations was centered at the Thr1Oγ with a radius of 15 Å. Covalent docking calculations were performed according to the following settings: CHEMPLP scoring function, 500 genetic algorithm (GA) runs, and 100% search efficiency. The boron atom of bortezomib was set as the link atom to covalently bind to the hydroxyl group oxygen of Thr1. Finally, the scores were ranked, and the results were visually analyzed. We selected the best 10 docking solutions based on the scoring function.

### 2.6 Key interaction determination

Protein and ligand were saved in PDB format with the MOE 2019.0102 software. The Python source code of the Protein-Ligand Interaction Profiler (PLIP) web server (Adasme et al., 2021) was installed locally, and ligand interactions were determined. A heatmap was generated with Seaborn (Waskom, 2021) and Matplotlib (for visualization with Python) (Hunter, 2007). The data analysis workflow was assembled using the Jupyter Notebook platform (Kluyver et al., 2016).

## 3 Results and discussion

**FIGURE 5:**
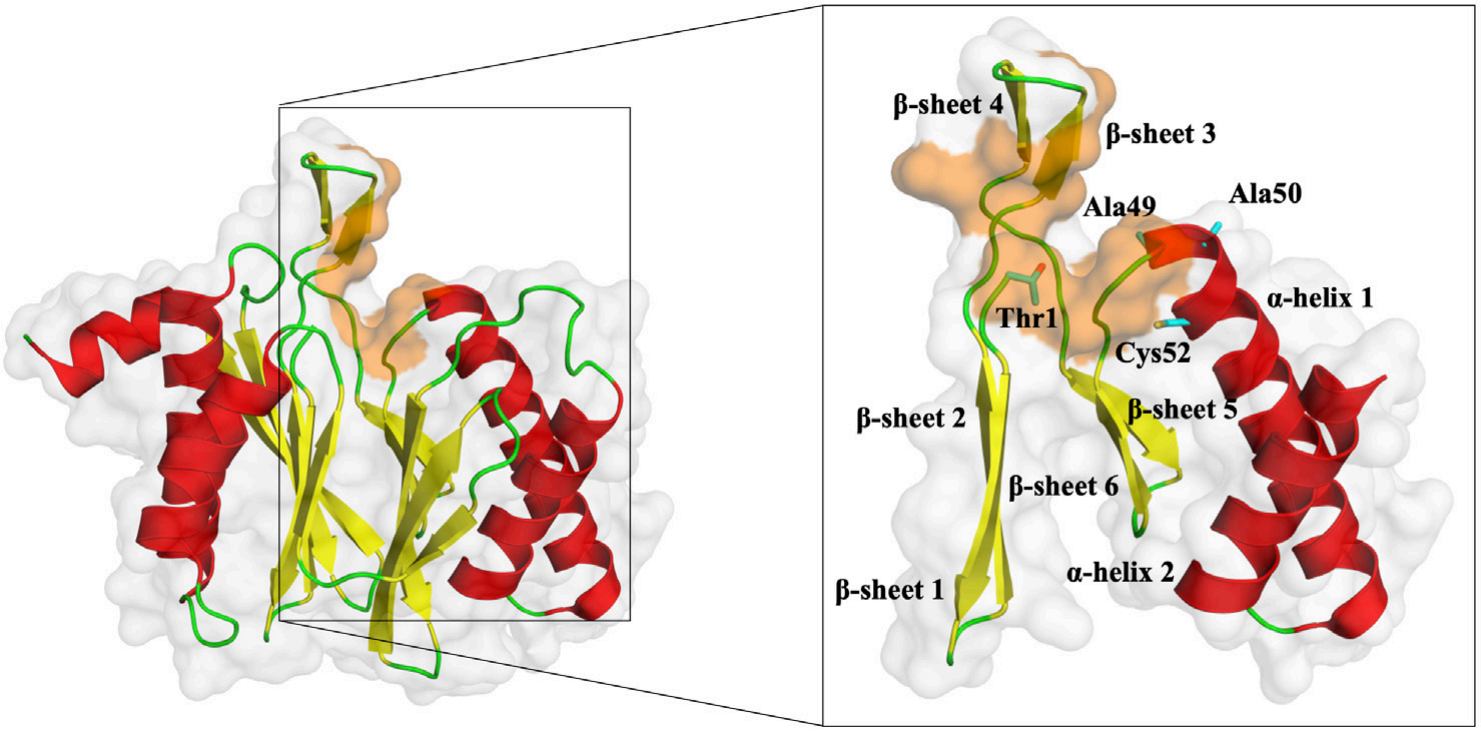
Representation of the β5 subunit, with emphasis on the catalytic pocket in orange. Part of the β5 subunit is represented by its secondary structure: β-sheet 1 (from residue 1–9), β-sheet 2 (from residue 10–18), β-sheet 3 (from residue 19–23), β-sheet 4 (from residue 24–29), β-sheet 5 (from residue 34–38), β-sheet 6 (from residue 39–47), α-helix 1 (from residue 49–70) and α-helix 2 (from residue 76–88). In sticks, we have also represented the N-terminal of the protein (Thr1), where typically 20S proteasome inhibitors covalently bind, Ala49, Ala50, and Cys52 (placed at the end of the β-sheet 3 and beginning of α-helix 1, respectively). This figure was built using PyMOL software (Schrödinger, 2023).

**FIGURE 6:**
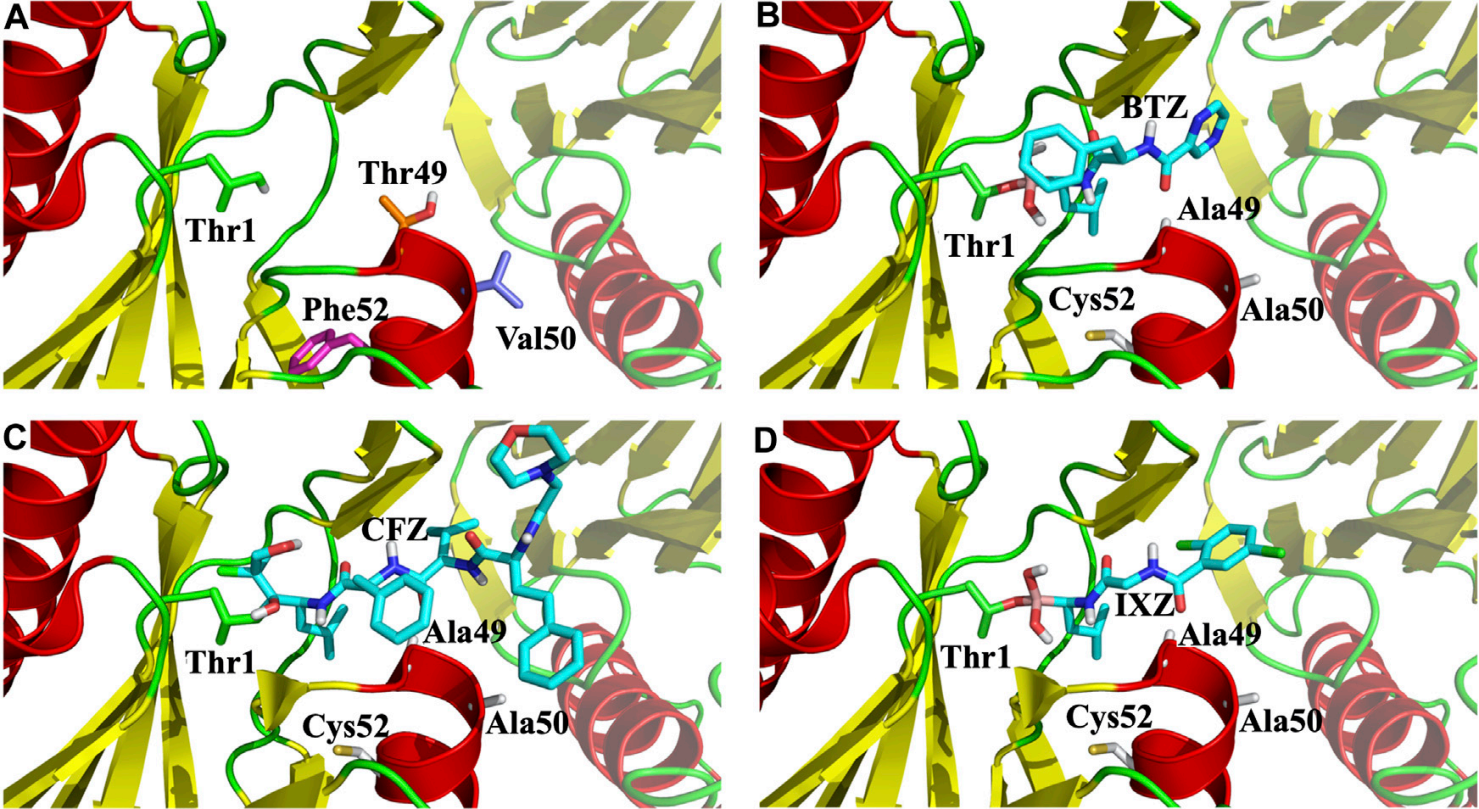
Zoom of the CT-L catalytic site of 20S proteasome. **(A)** Side chains of the three mutated residues and the N-terminal Thr1 are marked with sticks; **(B)**, **(C)**, and **(D)**, X-ray binding pocket positions of bortezomib (BTZ), carfilzomib (CFZ) and ixazomib (IXA), respectively. All figures show the β5 and β6 subunits as cartoon, with β6 being faded out.

Although in the available crystallographic structures of the 20S proteasome, the entire 20S core particle is available, in this work, we only simulated the two β-rings (Figure 1). In these two rings, one can find the region of interest of this work (the β5 subunit, namely, the CT-L pocket), guaranteeing at the same time the structural integrity of the protein core and a low computational cost of the simulations. As stated above, we focused our analysis on the stability of the β5 subunit in its WT form and three different mutants (Ala49Thr, Ala50Val, and Cys52Phe) due to their link to acquired resistance to various proteasome inhibitors. As seen in Figure 5, our mutational studies focused on the CT-L binding site region and are expected to influence inhibitor binding. However, the extent of structural changes associated with these mutants is still not fully understood and, with this study, we aim to gather more structural information on their impact, before evaluating the influence in the binding of bortezomib. According to structural modeling analysis, the mutant residues in this study, Ala49, and Ala50 are placed at the top of α-helix 1 (Figures 5, 6) suggesting their structural involvement in the enclosing of the CT-L active site. Regarding Cys52, one can see that this residue is placed in the middle of the same α-helix 1. Although it is not directly on the surface of the pocket, the mutation to a residue with different chemical properties is expected to impact the α-helix 1 stability, with consequent implications on the shape of the catalytic pocket.

### 3.1 Conformational analysis of the stability of the β5 subunit

To evaluate the impact of the three mutations on the dynamics of the 20S proteasome β5 subunit, we started by analyzing the root-mean-square deviation (RMSD) of the β-rings from the β5 subunits for the WT and studied mutants, using all replicate MD simulations.

**FIGURE 7:**
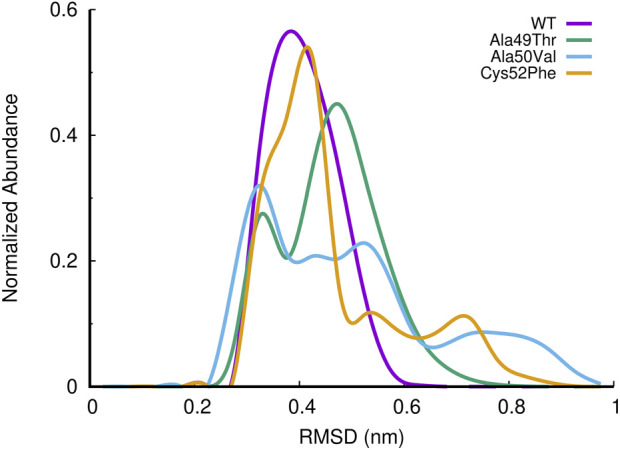
Histogram of the normalized abundance of the distribution of the RMSD of the c-alpha atoms of the β5 subunits for the WT, Ala49Thr, Ala50Val and Cys52Phe simulations. RMSD values were calculated taking as reference, the initial conformation of the system of the first replicate for each simulated system (only equilibrated regions of the simulations were considered).

In Figure 7, we have represented the RMSD distribution collected from the equilibrated parts of the different simulation systems in the form of the abundance of normalized RMSD histograms (Figure 7). The RMSD distribution of the WT system shows a profile resembling a perfect sigmoid, with a peak at around 0.4 nm of RMSD. On the other hand, all mutated systems show distinct and simultaneously, different profiles concerning the WT system, while visiting higher RMSD conformational states. A more detailed analysis of Ala49Thr mutant RMSD results revealed two major population sets of conformations found at around 0.3, (in a low degree) and 0.5 nm of RMSD (most populated conformation). Regarding Ala50Val simulations we can observe that this mutated simulated system visited a spread of conformations, ranging from 0.3 to 1 nm of RMSD concerning the reference stated, with clear peaks of conformational populations at 0.3 (most populated), 0.5, and 0.8. For simulations of the Cys52Phe mutant, we can observe a similar abundance profile of RMSD when compared to the WT system, with a major conformational peak observed around 0.4 nm of RMSD. However, this simulated system visits additional conformational states, up to values of around 0.9 RMSD, concerning the reference structure. All the mutated systems compared to the WT visited a higher conformational state, which can be related to changes in structural aspects of the binding pocket of the β5 subunit.

**FIGURE 8:**
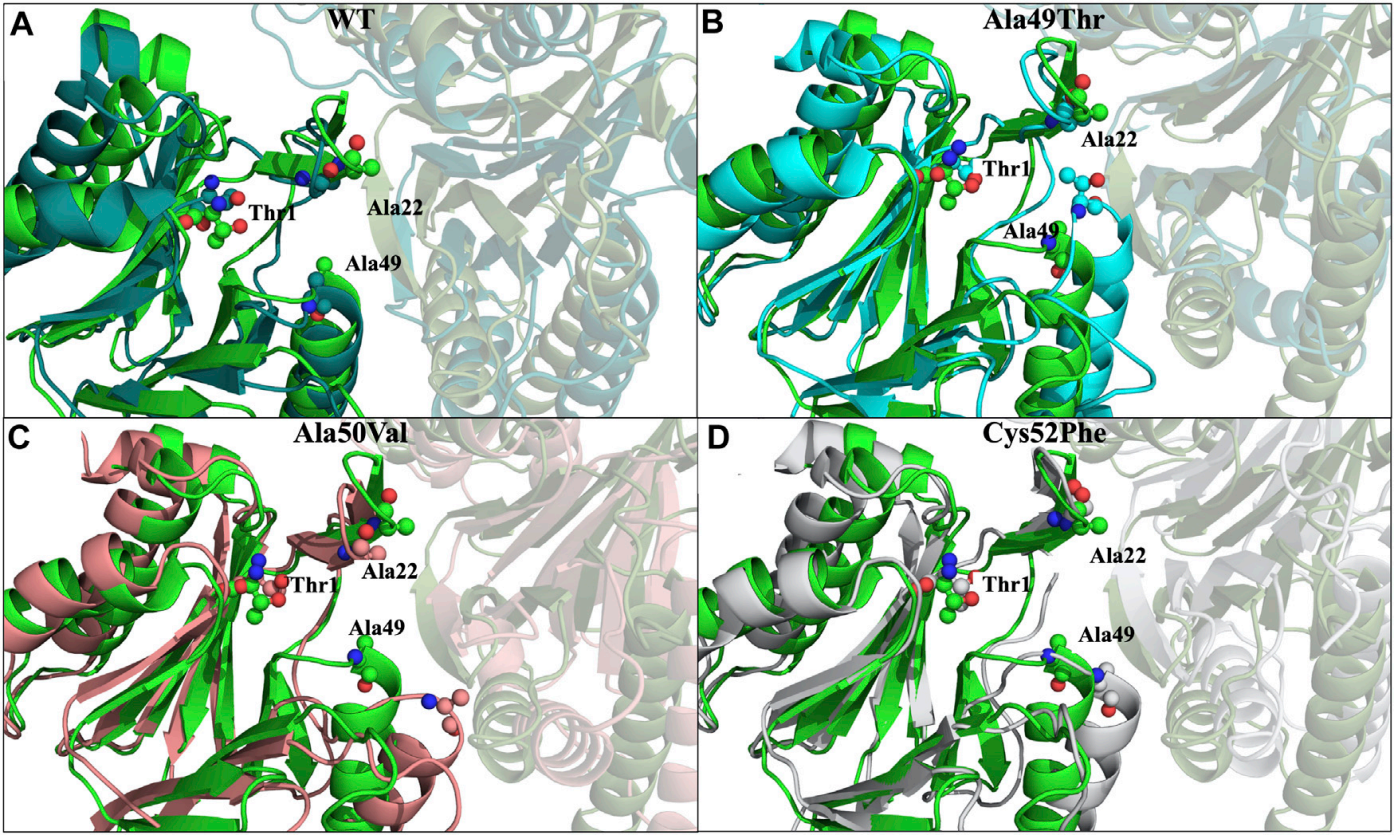
Most representative conformations of the different simulated systems. In light green we have represented the crystallographic structure (PDB code: 5LE5) of the β5 subunit, while the β6 subunit is represented as a faded cartoon. **(A)** In dark green we have represented the most populated conformation found in the simulations of the WT system, with a RMSD of 0.4 nm in respect to the crystallographic structure; **(B)** In cyan we have represented the most populated conformation found in the simulations of the Ala49Thr mutant, with a RMSD of 0.5 nm; **(C)** In light pink we have represented the most populated conformation found in the simulations of the Ala50Val mutant, with a RMSD of 0.3 nm; **(D)** In grey we have represented the most populated conformation found in the simulations of the Cys52Phe mutant, with a RMSD of 0.4 nm. RMSD values were calculated taking as reference, the initial conformation of the system of the first replicate for each simulated system.

If we analyze in detail the extracted representative conformations for the most populated peaks in each simulated system (Figure 8), we can structurally understand in detail which conformational changes were responsible for the observed differences.

Comparing the conformation of the β5 subunit in the crystal structure with the most populated conformation identified in the replicate MD simulations of the WT form of the proteasome, we observe a high general similarity between them, with the main differences arising from small loop movements and amino acid side-chain arrangements (Figure 8A).

Regarding the conformation extracted from Ala49Thr simulations with an RMSD around 0.5 nm (Figure 8B), the substitution of an alanine by a threonine appears to push α-helix 1 (Table 1) towards the β6 subunit, leading to a different structural arrangement that affects the shape of the CT-L binding pocket.

**TABLE 1:** Angle between α-helix 1 and α-helix 2 of β5 subunit.

Angle between α-helix 1 and α-helix 2 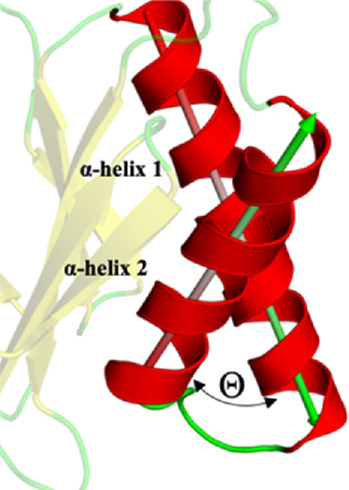				
X-ray	WT	Ala49Thr	Ala50Val	Cys52Phe
129.45°	133.14°	130.09°	131.25°	125.23°

In what concerned the results obtained from the simulations of the Ala50Val mutant, several changes can be identified in the β5 subunit. In the conformation extracted with a RMSD value of around 0.3 nm (Figure 8C), one can find a high resemblance with the conformation observed in the crystal structure of the WT protein. The main difference lies at the top of α-helix 1, where the mutation was placed, where a clear reduction in the helix length is observed. Moreover, the β-sheet 6 before this helix (Figure 5) also becomes shorter, losing most of its secondary structure towards a more unstructured arrangement. These conformational changes carry consequences at the bottom of the CT-L binding pocket, influencing its shape.

With respect to the results obtained for the Cys52Phe mutation we identify the most populated conformation around 0.4 nm (Figure 8D) of RMSD. Our observations indicate that in this representative conformation, this mutation leads to an increase in the occupied volume in an enclosed region of the β5 subunit, promoting a rotation of the phenylalanine residue into the catalytic pocket, and pushing α-helix 1 away from the Thr1 residue (Table 1). As shown in Figure 8D, the Phe52 residue turns towards the β6 subunit, with an associated unfolding of residues 48 to 52 (top of α-helix 1), affecting the shape of the binding pocket of the CT-L catalytic site. The less populated conformations obtained during the simulations are available in the (Supplementary Figure S1).

The secondary structure calculated using the dictionary of secondary structure (DSSP) (Kabsch and Sander, 1983; Touw et al., 2015) for the WT β5 subunit shows an average of 0.27 of β-sheet, 0.33 of α-helix, and 0.2 of coil. Overall, all the remaining analyzed mutant systems show very similar secondary structure variation profiles, indicating that despite the observed RMSD profiles, no significant structural variations affecting the secondary structure of these systems are observed.

**FIGURE 9:**
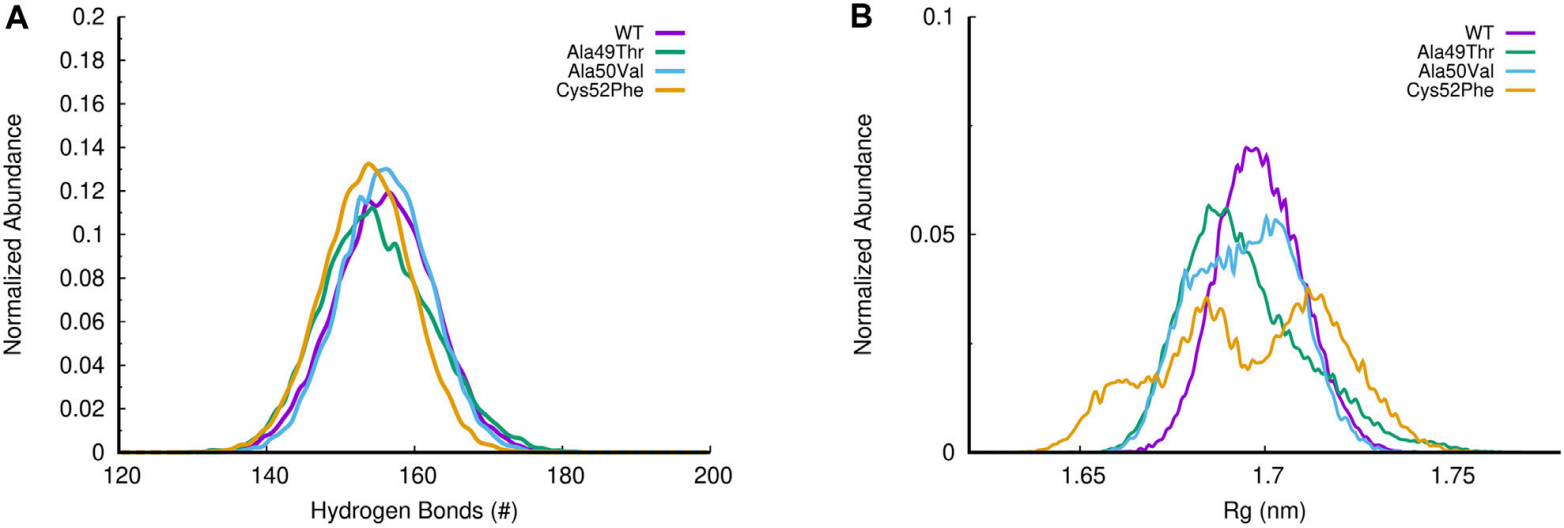
**(A)** Histogram of H-bonds distribution for all simulated systems; **(B)** Evaluation of the radius of gyration (R_*g*_) in all β5 subunit simulations.

To evaluate the compactness of the β5 subunit, we can evaluate simultaneously the number of hydrogen bonds (H-bonds) and the radius of gyration (Rg) of each simulated system. As shown in Figure 9A, the different mutations evaluated do not significantly impact the number of H-bonds established within the β5 subunit. However, when we analyze the radius of gyration, we notice that despite both Ala49Thr and Ala50Val systems showing a very similar profile to that observed in the WT protein, the Cys52Phe mutant shows (Figure 9B) distinct compactness of the sampled conformational distribution. In the simulation of the former mutated system, it seems that the structure fluctuates between two conformational clusters, one with a more compact (with a radius of gyration around 1.68 nm), and another one with a less compact conformation (with higher values of the radius of gyration around 1.72 nm).

### 3.2 Chymotrypsin-like binding pocket (β5 subunits) of the 20S proteasome

Focusing our analysis on the catalytic pocket of the β5 subunit, we can see that this pocket is mainly shaped by three regions in the β5 subunits: the terminus of β-sheet 1 and 2, respectively; the region below β-sheet 3; the beginning of α-helix 1 and the beginning and end of the β-sheet 5 and 6, respectively. The cleft resulting from these boundary regions makes the concavity of the conformation of the 20S proteasome perfect to accommodate stable protein-ligand interactions. To evaluate the effect of the studied mutations on the CT-L binding region of the β5 subunit of the 20S proteasome we have analyzed the distance between the three key residues in this region, which delimits the entire binding pocket: Thr1, which is the N-terminal where the covalent proteasome inhibitors bind to the protein; Ala22, a residue placed in the middle of the loop between β-sheet 3 and β-sheet 4 (Figure 5 for details); and, finally, Ala49, which is one of the mutated residues, and found on top of the α-helix 1.

**FIGURE 10:**
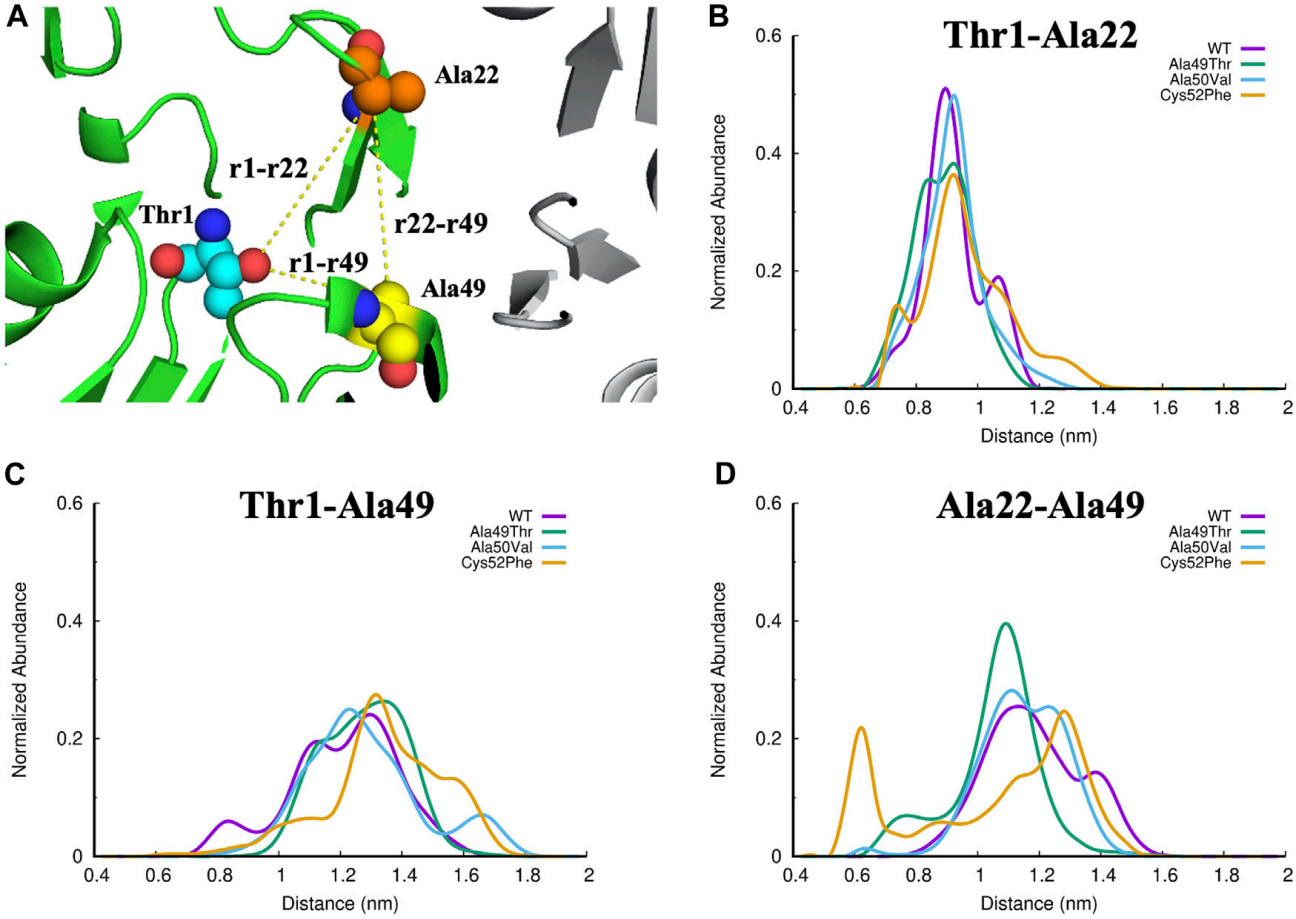
**(A)** Identification of the three regions of interest in the CT-L active site of the proteasome. The Thr1, Ala22 and Ala49 atoms are represented as small spheres, and the yellow lines represent the distances evaluated: **(B)** Thr1-Ala22, **(C)**Thr1-Ala49, and **(D)** Ala22-Ala49.

An accurate analysis of the abundance of the different distances calculated in the equilibrated parts of the simulations of each system (Figure 10) indicates that the distances Thr1-Ala49 and Ala22-Ala49 show significant changes when compared with the distances between Thr1-Ala22. This suggests that the region where Ala49 is located is more susceptible to changes in its conformation in all the evaluated systems. As can be seen in Figures 10C,D, the Cys52Phe mutant is the most disruptive mutant of the ones tested since the abundance of the distance Ala22-Ala49 shows a much different profile when compared to all other simulated systems, suggesting in a first analysis, that this mutant promotes the greatest conformational changes from the evaluated systems, in the configuration of the CT-L binding site. As mentioned above, the inclusion of a more hydrophobic and larger residue (a cysteine is mutated by phenylalanine) forces significant structural changes in this region of the protein, changing both the shape and volume of the pocket.

Comparing the volume of the catalytic pocket in the most populated conformations obtained from the MD simulations for each set of simulations, we can see a wide range of volume. As seen in Table 2, concerning the crystal structure, we can see that a slightly higher volume of the catalytic pocket of β5 subunit was determined in the representative conformation of the WT simulation, when compared to the WT crystallographic structure (775 Å3 compared to 531 Å3). The observed difference is expected since in the MD simulations there is no inhibitor to constrain the volume of the pocket, and the observed results occur due to a structural relaxation of this region of the protein, leading to changes affecting its shape and volume. In the mutated system Ala49Thr the determined volume of the catalytic pocket of the representative conformation analyzed was 920 Å3, the highest pocket value identified in all systems under study. Regarding the representative conformation Ala50Val, the determined pocket volume was 742 Å3, which is a value very similar to the one determined for the WT system. Finally, for the representative conformation of the Cys52Phe system, the determined volume of the catalytic pocket was the lowest of all systems, indicating that the mutation of Cys52 to a larger hydrophobic residue significantly decreased the size of the pocket, up to a level that could indicate its collapse. The results focused on the analysis of the pocket volumes for each one of the simulated systems can be correlated with the previously described results focused on the analysis of the distances between key residues (Figure 10). From the analysis of both properties, we can conclude that the mutation Cys52Phe has a consistently higher impact on the structure stability and dynamics of the binding pocket.

**TABLE 2:** Volume of the catalytic pocket of β5 subunit (20S proteasome). Volumes were calculated using POVME software considering only the most populated conformations of each set of simulations reported in Figure 7.

System	RMSD (nm)	Volume (Å^3^)	Ratio
X-ray		531	
WT	0.4	775	1
Ala49Thr	0.5	920	1.19
Ala50Val	0.3	742	0.96
Cys52Phe	0.4	672	0.87

**FIGURE 11:**
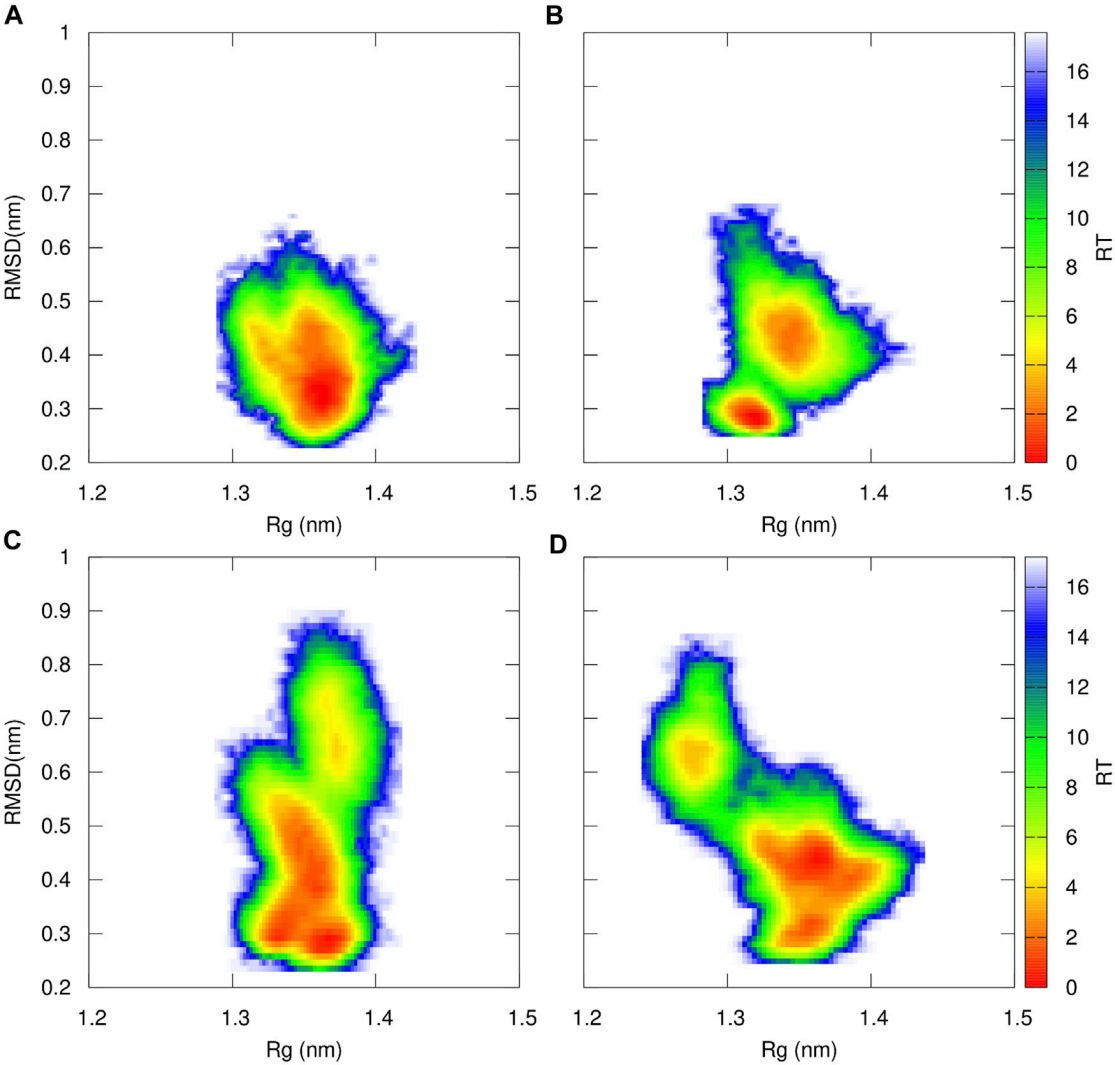
Free Energy Profiles for WT **(A)**, Ala49Thr **(B)**, Ala50Val **(C)** and Cys52Phe **(D)** of the catalytic region of the β5 subunit of the 20S proteasome that delimits the catalytic pocket (residues 1 to 60–Figure 5 for structural details) using RMSD and R_*g*_ as structural coordinates. Both RMSD and R_*g*_ were calculated using GROMACS software tools.

To further characterize the proteasome’s binding pocket configurations of the β5 subunit, we calculated the Free Energy conformational profile for the WT, Ala49Thr, Ala50Val, and Cys52Phe based on the RMSD and Rg of the residues delimiting the catalytic pocket (residues 1–60) (Figure 11). In the WT simulations, one major conformational region is prevalent, as previously concluded from the histograms shown in Figure 7. However, this type of conformational profile is not observed in any of the mutant systems. If we focus on the results of Ala49Thr, despite the free energy profile being similar to the one observed for the WT system, we can identify two main basins, representing two distinct sets: one at around 0.3 nm of RMSD with a more “closed” conformation and another at 0.5 nm with a more “open” conformation (with a higher volume–table SM 1). Regarding the results obtained for the Ala50Val system, we can identify that a broader conformational space is populated with respect to the WT system, with three main conformational regions found at 0.3, 0.5, and 0.8 nm values of RMSD, each one with small differences on the observed Rg values. These small, but significant distances in the Rg, indicate the existence of open and closed configurations, consistent with the determined volumes shown in Table SM 1. The analysis of the Free energy profiles of the Cys52Phe mutant simulations, clearly indicates that this system is the one with a more dispersed configurational profile when compared to all the other simulated systems. From Figure 11D, we can identify two major configurational regions: one comprising two conformational results (RMSD 0.4 and 0.5 nm) with a more open configuration and, therefore, with higher volumes (Table 2), and a second one with higher RMSD values but with a more closed configuration (lower value of Rg) and therefore with a lower volume (Table 2). These results indicate that a good correlation between Rg and volumes of pockets is obtained.

### 3.3 Impact of mutations on β5-β6 interactions

**FIGURE 12:**
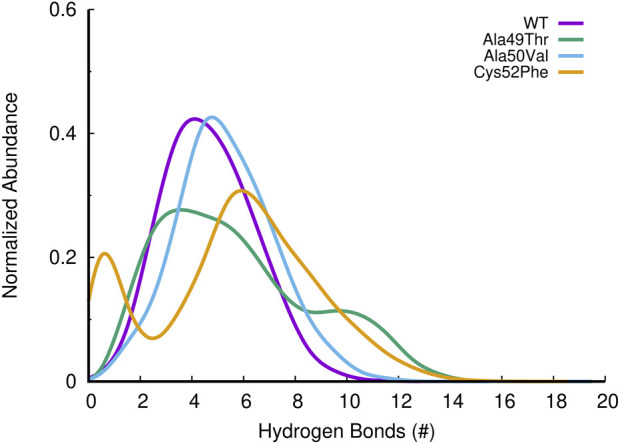
Histogram of the abundance of hydrogen bonds found between β5 and β6 subunits of the 20S proteasome, for the different simulated systems.

The β6 subunit of the 20S proteasome contributes to the structure integrity and stability of the catalytic pocket present in the β5 subunit. Analyzing the interactions between these two subunits can further help us understanding the influence of the mutations on the binding pocket. To evaluate the effect of the different mutations on the interaction between the β5 and β6 subunits of the 20S proteasome, we assessed the number of hydrogen bonds established between these two subunits during the simulations. As can be seen in Figure 12, the number of hydrogen bonds established between the two subunits in the Ala50Val mutation simulations is very similar when compared to the results obtained from the WT simulations. However, Ala49Thr mutation appears to promote some structural changes that significantly affect the interactions between β5 and β6 subunits. As shown in Figure 12, two major conformations are predominant: one in which the average number of hydrogen bonds is around 4–6 and another configuration with approximately 9–12 H-bonds. When analyzed together with the results discussed in the previous sections, we can conclude about the existence in the Ala49Thr system of a higher packing/interaction between the two subunits, most probably related to an approximation of α-helix 1 of the β5 subunit to the β6 subunit. Regarding the Cys52Phe system, we can observe a higher variance in the number of hydrogen bonds. These results are consistent with the Rg vs. RMSD plots of the β5 subunit previously analyzed, where it is possible to identify high conformational variability in the Cys52Phe simulations. Similarly, to what could be observed in the previous sections, this mutation seems to affect the shape of the binding pocket, influencing the interaction of the β5 and β6 subunits.

### 3.4 β5 molecular docking

After studying the impact of these mutations on the structural stability and dynamics of the binding pocket of the β5 subunit, we aim to understand how these mutations can affect the binding interactions between the β5 subunit and a known proteasome inhibitor, bortezomib. The extensive research on bortezomib over the years offers a solid foundation for further investigation. The understanding of its mechanism of action is relatively more comprehensive compared to newer drugs, providing a clearer starting point for studying the effects of point mutations on drug binding and efficacy. Investigating bortezomib can provide insights that are potentially applicable to newer proteasome inhibitors.

**FIGURE 13:**
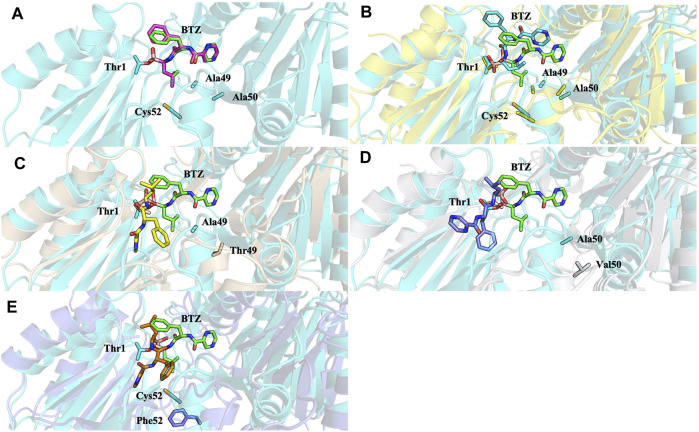
Superposition of the bortezomib docking poses of X-ray WT with docking results for: **(A)** bortezomib in docking validation, **(B)** WT, **(C)** Ala49Thr, **(D)** Ala50Val, and **(E)** Cys52Phe mutations with crystal structure 5LF3 (the crystallographic bortezomib is represented in green).

**FIGURE 14:**
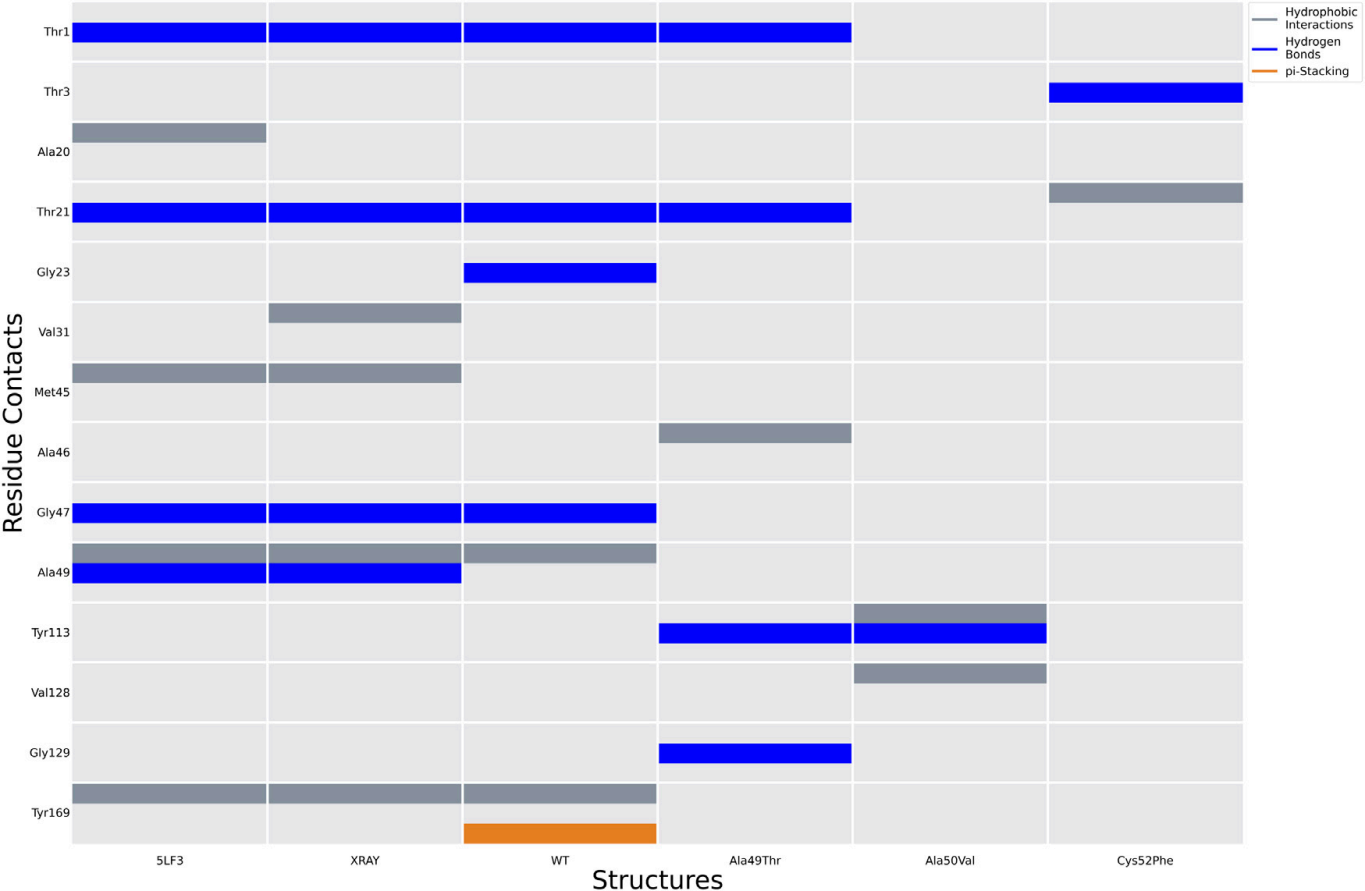
Ligand interactions established between bortezomib with the proteasome in the crystal structure 5LF3 and docking calculations performed using selected snapshots extracted from MD simulations of the WT and Ala49Thr, Ala50Val, and Cys52Phe mutants.

**FIGURE 15:**
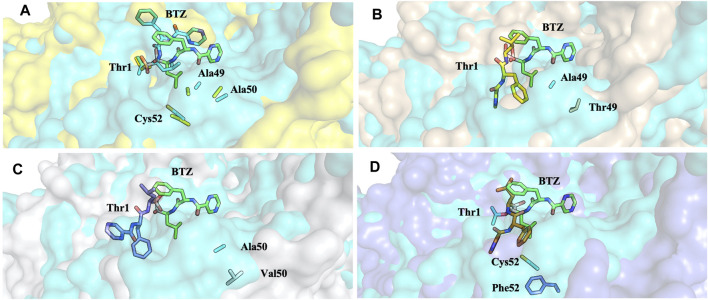
Superposition of the bortezomib docking poses of WT **(A)**, Ala49Thr **(B)**, Ala50Val **(C)**, and Cys52Phe **(D)** mutations with crystal structure 5LF3 (the X-ray BTZ is represented in green). β5-β6 respective protein’s surfaces.

Analysis of molecular docking calculations of the binding mode of bortezomib at the CT-L active site, when compared with the 5LF3 crystal structure, allows the observation of significant changes in the binding poses at the mutated structures and, consequently, in the protein-ligand (non-covalent) interactions that are formed (Figures 13–15). Our molecular docking protocol was initially used to try to reproduce the binding pose of bortezomib determined at the X-ray structure. As can be seen in Figure 13A, we were able to almost reproduce the binding of this compound exactly at the evaluated binding site. By analyzing the complex determined at the crystallographic structure, we could identify several interactions between bortezomib and Thr1 (H-bond), Ala20 (hydrophobic interaction), Thr21 (H-bond), Met45 (hydrophobic interaction), Gly47 (H-bond), Ala49 (H-bond and hydrophobic interactions), and Tyr169 (hydrophobic interaction) from the proteasome. Comparing the pose determined in the crystallographic structure and the ones obtained from our docking protocol we can observe the rotation of single bonds upon the docking of bortezomib in the WT structure (Figure 13B), driving a decrease in the interactions established with the protein (we can highlight the interaction with Thr1, Thr21, and Ala50).

Molecular docking calculations of bortezomib in the three mutant structures–Ala49Thr, Ala50Val, and Cys52Phe (Figures 13C–E, respectively)–strongly suggest that the presence of these single mutations alters bortezomib pose in the CT-L active site, impairing interactions with the S1 pocket, and hindering catalytic activity. Steric hindrance (due to bulkier side chains in the mutant residues) leads to substantial torsions resulting in significant changes in the binding poses (e.g., in Ala50Val mutation, the pyrazine ring of bortezomib is about 12 Å from its position in the crystal structure) and consequently, a decrease in the number of key interactions of the ligand: the Ala49Thr mutation still allows the interaction of bortezomib with Thr1 (H-bond) and Thr21 (H-bond). However, in the Ala50Val mutation, bortezomib fails to interact with any residue usually enrolled for catalytic activity. In the Cys52Phe mutant, only the interaction with Thr21 is maintained, although switching from an H-bond to a hydrophobic interaction.

Mutation of the non-polar Ala49 to the polar threonine causes a steric clash with the bortezomib inhibitor and additionally with the proteasome’s β6 subunit, suggesting that the compounds failed to access the modified pocket preventing the binding.

## 4 Conclusion

Clinical resistance to proteasome inhibitors is a complex and challenging issue, influenced by multifactorial mechanisms such as mutations. To elucidate these resistance mechanisms and understand the structural changes that occur in β5 subunit due to the emergence of mutations, here we report a computational study, using MD simulations and molecular docking, focused exclusively on three single mutations (Ala49Thr, Ala50Val, and Cys52Phe) which are involved in the binding of proteasome inhibitors (e.g., bortezomib) to the CT-L active site. Ala49 and Ala50 mutations were selected given their crucial location and potential impact on shaping and enclosing the active site. Cys52 is located in the middle of α-helix 1 in the N-terminal and, although not directly on the pocket surface, its mutation to a more hydrophobic and bulkier residue (Phe) has implications for the stability of α-helix 1 and hence the conformation of the binding pocket. The mutations were analyzed for their impact on proteasome conformation, functionality, and binding.

Trajectories obtained from the MD simulations were analyzed considering the RMSD, distances, H-bond contacts, binding pocket volume, and β5-β6 interactions for the WT and the three mutant variants. In addition to comparing the stability of the β5 variants, this study also explored the factors that may contribute to their stability and binding to inhibitors. Volume and “druggability/affinity” of mutants binding pockets by using bortezomib binding as an illustrative example were investigated. All mutant systems exhibit a greater conformational variability for the β5 subunit (RMSD) when compared to the WT system. These significant differences are the result of side-chain rearrangements and small displacements of the protein backbone. Significant changes were observed in the distances between Thr1-Ala49 and Ala22-Ala49, indicating that the different amino acid substitutions are more susceptible to conformational changes in the region where Ala49 is placed. Moreover, the substitution of Cys52 for a more hydrophobic and larger residue such as phenylalanine, promotes changes in the shape and volume of the pocket, leading to significant structural changes in the closest protein regions such as the CT binding pocket. Furthermore, MD simulations showed that the volume of the catalytic pocket also changes with the analyzed mutations. This suggests that future drug design efforts should account for these conformational changes to ensure effective binding. Additionally, in the Ala50Val system, a similar number of hydrogen bonds is established between the β5 and β6 subunits, while in the Ala49Thr mutation, the interaction between these subunits is affected (two major conformations are predominant: one with an average number of hydrogen bonds around 4 to 6, and another one with 9–12 hydrogen bonds), since a higher packing/interaction between the two subunits is observed. These observations are probably related to an approximation of α-helix 1 of the β5 subunit to the β6 subunit. Regarding the Cys52Phe mutation, a higher variance of hydrogen bonds was observed confirming the higher conformational variability in the Cys52Phe simulations. Changes in hydrogen bonding patterns, particularly in the Ala49Thr mutation, indicate altered interactions between the β5 and β6 subunits. This highlights the need for inhibitors that can adapt to these new interaction patterns.

Molecular docking calculations showed that the three mutations affect the binding to the CT-L active site, namely, through changes that occur in the S1 pocket and, consequently, modify the interaction pattern of bortezomib with the CT-L active site residues. Bulkier side chains leading to steric hindrance, fewer hydrogen bonds, fewer interactions with relevant residues, and thus the possibility of rotation of bortezomib, lead to a different position at the active site. Considering all these results, one can conclude that the Ala49Thr, Ala50Val, and Cys52Phe mutations change the conformational structure of the 20S proteasome β5 subunit pocket, suggesting a significant influence on the resistance mechanisms associated with the therapeutic use of bortezomib. The insights gained can guide the development of more potent and selective drugs, capable of overcoming resistance mechanisms like those seen with bortezomib, thereby enhancing therapeutic efficacy in treating conditions like MM and other cancers where proteasome inhibitors are employed.

## Data Availability

The datasets presented in this study can be found in online repositories. The names of the repository/repositories and accession number(s) can be found in the article/Supplementary Material.
