# Supplementary material for: Decoding the secrets: how conformational and structural regulators inhibit the human 20S proteasome
Source: Front Chem. 2024 Jan 8;11:1322628. doi: 10.3389/fchem.2023.1322628 (PMC10801056; doi:10.3389/fchem.2023.1322628)
Supplement: Supplementary file 1 [file DataSheet1.PDF]

Decoding the Secrets: How Conformational and Structural Regulators Inhibit the Human 20S Proteasome

Pedro Fernandes[1,2,3], Romina A. Guedes[1,2,3], Bruno L. Victor[4], Jorge A. R. Salvador[1,2*], Rita C. Guedes[3*]

[1]Laboratory of Pharmaceutical Chemistry, Faculty of Pharmacy, University of Coimbra, 3000-548 Coimbra, Portugal

[2]Center for Innovative Biomedicine and Biotechnology (CIBB), Center for Neuroscience and Cell Biology (CNC), University of Coimbra, 3004-504 Coimbra, Portugal

[3]Research Institute for Medicines (iMed.ULisboa), Faculdade de Farmácia, Universidade de Lisboa, 1649-003 Lisboa, Portugal

[4]BioISI─Biosystems & Integrative Sciences Institute, Faculty of Sciences, Universidade de Lisboa, 1749-016 Lisboa, Portugal

*** Correspondence:**
Corresponding Author: salvador@ci.uc.pt; rguedes@ff.ulisboa.pt;

Keywords: 20S Proteasome Inhibitors; Resistance; Mutations; Molecular Dynamics; Molecular Docking

Supplementary Material

1 Supplementary Data

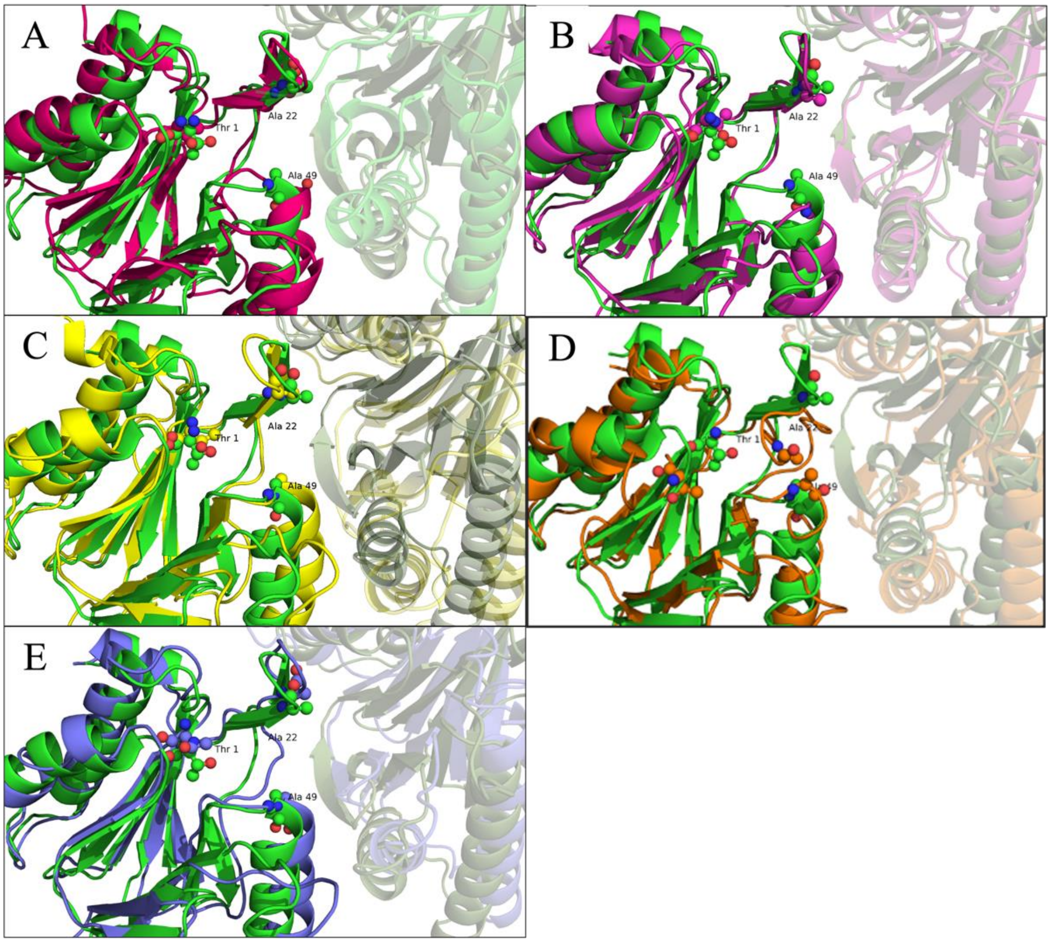

Supplementary Figure 1. (A) – In light green we have represented the crystallographic structure, and in magenta we have represented the most populated conformation found in the simulations of the Ala49Thr mutant, with a RMSD of 0.3 nm in respect to the crystallographic structure; (B) – In light green we have represented the crystallographic structure, and in pink we have represented the most populated conformation found in the simulations of the Ala50Val mutant, with a RMSD of 0.5 nm in respect to the crystallographic structure; (C) – In light green we have represented the crystallographic structure, and in yellow we have represented the most populated conformation found in the simulations of the Ala50Val mutant, with a RMSD of 0.8 nm in respect to the crystallographic structure; (D) – In light green we have represented the crystallographic structure, and in orange we have represented the most populated conformation found in the simulations of the Cys52Phe mutant, with a RMSD of 0.5 nm in respect to the crystallographic structure; (E) – In light green we have represented the crystallographic structure, and in blue we have represented the most populated

conformation found in the simulations of the Cys52Phe mutant, with a RMSD of 0.7 nm in respect to the crystallographic structure.

Table SM 1 - Volume of the catalytic pocket of the β5 20S proteasome. Volumes were calculated using POVME software, for each of the most representative conformations of each set of simulations reported in Figures 8 and S1.

Structure		Volume of the pocket ($Å^3$)
X-ray	X-ray	531
	0.4 nm of RMSD	775
Ala49Thr	0.3 nm of RMSD	343
	0.5 nm of RMSD	920
Ala50Val	0.3 nm of RMSD	742
	0.5 nm of RMSD	929
	0.8 nm of RMSD	719
Cys52Phe	0.4 nm of RMSD	672
	0.5 nm of RMSD	584
	0.7 nm of RMSD	426
